# Vasopressin in cardiogenic shock: pathophysiology, clinical evidence, and therapeutic perspectives

**DOI:** 10.1093/eschf/xvag053

**Published:** 2026-02-13

**Authors:** Simone Filomia, Marco Giuseppe Del Buono, Gianluigi Saponara, Alessia d’Aiello, Daniela Pedicino, Francesco Moroni, Antonio Abbate, Tommaso Sanna

**Affiliations:** Department of Cardiovascular Sciences, Fondazione Policlinico Universitario A. Gemelli IRCCS, L.go A. Gemelli, Rome 1–00168, Italy; Department of Cardiovascular Sciences, Fondazione Policlinico Universitario A. Gemelli IRCCS, L.go A. Gemelli, Rome 1–00168, Italy; Department of Cardiovascular Sciences, Fondazione Policlinico Universitario A. Gemelli IRCCS, L.go A. Gemelli, Rome 1–00168, Italy; Department of Cardiovascular Sciences, Fondazione Policlinico Universitario A. Gemelli IRCCS, L.go A. Gemelli, Rome 1–00168, Italy; Department of Cardiovascular Sciences, Fondazione Policlinico Universitario A. Gemelli IRCCS, L.go A. Gemelli, Rome 1–00168, Italy; Berne Cardiovascular Research Center, University of Virginia, Charlottesville, VA, USA; Berne Cardiovascular Research Center, University of Virginia, Charlottesville, VA, USA; Department of Cardiovascular Sciences, Fondazione Policlinico Universitario A. Gemelli IRCCS, L.go A. Gemelli, Rome 1–00168, Italy; Department of Cardiovascular and Respiratory Sciences, Catholic University of the Sacred Heart, Largo Francesco Vito, 1, Rome 00168, Italy

**Keywords:** Cardiogenic shock, Vasopressors, Vasopressin, Mixed shock, Tachyarrhythmias, Haemodynamic support

## Abstract

Vasopressin is a non-adrenergic vasopressor, commonly used in distributive shock, that may offer therapeutic benefits in selected phenotypes of cardiogenic shock.

This review presents a comprehensive synthesis of its pharmacologic mechanisms, haemodynamic effects, clinical applications, and potential adverse outcomes in this complex setting. We delineate clinical scenarios in which vasopressin may be advantageous, critically appraise the current evidence, and support a physiology-guided, individualized approach to treatment.

Patient profiles that may particularly benefit include advanced or mixed cardiogenic shock with low systemic vascular resistance, tachyarrhythmia-related shock, post-cardiac arrest shock, acute right ventricular failure, mitral stenosis, prosthetic mitral valve thrombosis, dynamic left ventricular outflow tract obstruction, Takotsubo syndrome, and severe aortic stenosis. In these settings, vasopressin’s non-adrenergic mechanism of action may improve perfusion pressure while mitigating catecholamine-related adverse effects.

We also outline clinical scenarios of cardiogenic shock in which vasopressin use may be detrimental and discuss its implications in patients receiving mechanical circulatory support.

Finally, we highlight the need for prospective studies to optimize vasopressor selection in this high-risk and heterogeneous population.

## Introduction

Cardiogenic shock is a critical medical emergency characterized by a severe impairment in cardiac function, resulting in inadequate tissue perfusion and oxygen delivery that fails to meet the body’s metabolic demands. Despite significant advancements in diagnostic tools and therapeutic strategies, cardiogenic shock remains associated with high morbidity and mortality rates, posing an ongoing challenge for healthcare professionals.^1^

Its pathophysiology involves profound haemodynamic derangements, cellular metabolic dysfunction, and systemic inflammatory responses, which together create a vicious cycle that contributes to progressive multiorgan failure.^2^

Inotropes and vasopressors constitute the cornerstone of pharmacologic therapy, aiming to support the heart’s pumping capacity and ensure sufficient perfusion of vital organs. These agents are often complemented by mechanical circulatory support (MCS) devices.

Pharmacologic strategies must be tailored to the aetiology and phenotypic profile of cardiogenic shock, as no single therapeutic algorithm applies universally across all cases. Although inotropic and vasopressor agents are commonly employed in the management of cardiogenic shock, evidence supporting the use of a specific agent remains limited and largely inconclusive. Most available data derive from small randomized trials or observational studies with heterogeneous patient populations and endpoints. As a result, the choice of inotropes or vasopressors in clinical practice is frequently guided more by pathophysiological reasoning and individual haemodynamic profiles than by robust comparative effectiveness data.^3^

A non-adrenergic vasopressor such as vasopressin may represent a valuable therapeutic option in selected clinical scenarios. This review aims to synthesize the current evidence regarding the use of vasopressin in cardiogenic shock, with a focus on its pharmacological properties, haemodynamic effects, and potential adverse outcomes.

We also seek to identify the specific clinical contexts in which its use may be appropriate, in order to lay a foundation of knowledge and evidence that can support the design of future clinical studies.

## Mechanism of action of vasopressin

Vasopressin, also known as arginine vasopressin (AVP) or antidiuretic hormone (ADH), is a nonapeptide hormone synthesized by the supraoptic and paraventricular nuclei of the hypothalamus and subsequently released from the posterior pituitary gland, where it is co-secreted in equimolar amounts with copeptin—the C-terminal fragment of the AVP prohormone and a more stable surrogate biomarker of AVP release. AVP’s physiological actions extend beyond fluid homeostasis to key cardiovascular, renal, and neuroendocrine effects. Notably, AVP exerts potent vasopressor activity independent of adrenergic signalling, offering therapeutic value when adrenergic receptors are desensitized, catecholamine doses are maximized, further adrenergic stimulation would be harmful, or adrenergic blockade is present.

AVP exerts its diverse biological actions by binding to a family of G protein-coupled receptors (GPCRs), namely V1a, V2, and V3 (V1b) receptors, each coupled to distinct intracellular signalling pathways and exhibiting specific tissue distributions.^4^

V1a receptors are ubiquitously expressed on vascular smooth muscle cells across both the systemic and pulmonary circulations, and are also found on platelets, hepatocytes, and myocardial cells. Upon binding to these receptors, vasopressin activates Gq protein-coupled signalling pathways, leading to increased intracellular calcium concentrations and subsequent smooth muscle contraction. This signalling cascade results in potent vasoconstriction, manifesting as increased systemic vascular resistance (SVR) and elevated mean arterial pressure (MAP).

However, the vasoconstrictive effect on the pulmonary circulation appears to be significantly lower than in the systemic circulation,^5,6^ likely due to a reduced density of V1a receptors in the pulmonary vasculature, as well as vasopressin-induced endothelial nitric oxide production, which counteracts vasoconstriction.^7,8^ This selective vascular profile may confer a haemodynamic advantage by preserving right ventricular–pulmonary arterial coupling, particularly in patients with right ventricular dysfunction or severe pulmonary hypertension, where minimizing pulmonary vascular resistance is crucial.^9^

V2 receptors are located on the basolateral membrane of principal cells in the renal collecting ducts. AVP binding to V2Rs promotes translocation of aquaporin-2 water channels from intracellular vesicles to the apical membrane, thereby increasing water reabsorption into the bloodstream and resulting in antidiuresis and urine concentration, which helps preserve intravascular volume.

V3 receptors (V1b receptors in older nomenclature) are primarily found in the anterior pituitary gland, where AVP acts synergistically with corticotropin-releasing hormone (CRH) to stimulate the release of adrenocorticotropic hormone (ACTH), contributing to the body's neuroendocrine response to stress.

The clinically available formulation of AVP consists of an aqueous solution for continuous intravenous infusion, typically supplied at a concentration of 20 units/ml. It is diluted in normal saline or dextrose-containing solutions before administration. The standard dosing regimen in cardiogenic shock ranges from 0.01 to 0.03 units per minute. Unlike other vasopressors, vasopressin is generally administered at a fixed dose without titration, and doses above 0.04 units/min are avoided due to the increased risk of ischaemic complications.

Pharmacokinetically, vasopressin has a short plasma half-life of approximately 10 minutes. It is partially metabolized in the liver and kidneys, with renal excretion as the main route of elimination. Specific pharmacokinetic studies in patients with hepatic or renal dysfunction are lacking. Notably, vasopressin may retain its vasoconstrictive efficacy under acidotic or hypoxic conditions—common in advanced cardiogenic shock—more than catecholamines do.^10^

Terlipressin is a synthetic vasopressin analogue and prodrug with a prolonged half-life (3–6 h), permitting intermittent bolus dosing. It is more V1a-selective, producing potent splanchnic vasoconstriction. Indications include variceal bleeding and hepatorenal syndrome; it should not be used to treat shock.^11^

Desmopressin (DDAVP) is a V2-selective vasopressin analogue developed for central diabetes insipidus. In brain-dead organ donors, it limits polyuria and corrects hypernatremia, aiding organ preservation.^12^ By activating endothelial V2 receptors, it releases stored factor VIII and von Willebrand factor, hence its use in selected cases of von Willebrand disease (especially type 1).

## Adverse effects of vasopressin

Vasopressin’s potent vasoconstrictive action—primarily mediated via V1a receptors—can lead to significant adverse effects, particularly at higher doses or with prolonged administration.

Ischaemic complications are among the most concerning. Digital ischaemia has been reported in approximately 8–10% of patients, while mesenteric ischaemia may occur in up to 10–20%, especially when infusion durations exceed 48 h or in patients receiving high-dose therapy.^13,14^ Early recognition is crucial and should involve frequent monitoring of serum lactate levels, careful evaluation for new or worsening abdominal pain, distension, and signs of paralytic ileus. Peripheral perfusion should be regularly assessed by inspecting the extremities for cyanosis, mottling, or evolving skin changes.

Myocardial ischaemia, though relatively infrequent, may occur as a consequence of increased systemic afterload, particularly in patients with pre-existing coronary artery disease. However, available evidence does not suggest a significantly higher incidence compared to norepinephrine,^13^ even among patients with cardiogenic shock.^15^

Similarly, cardiac contractility does not appear to be markedly impaired by AVP at clinically used doses. Despite the theoretical disadvantage of increased afterload in failing hearts, AVP has been shown to raise MAP without significantly affecting cardiac index or pulmonary capillary wedge pressure.^15^ Some studies have even reported a potential mild positive inotropic effect at low doses, possibly mediated by cardiac V1a receptors.^16^ In contrast, high-dose AVP may reduce coronary blood flow and impair myocardial performance due to excessive vasoconstriction.^17^

In terms of arrhythmogenic potential, vasopressin is considered neutral, as it lacks both chronotropic and proarrhythmic effects. A large meta-analysis demonstrated a reduced incidence of atrial fibrillation in patients with distributive shock treated with AVP in addition to catecholamines, compared to catecholamines alone—likely due to its catecholamine-sparing effect.^18^

Hyponatremia is another documented adverse effect, occurring in approximately 1%–3% of patients,^14^ due to V2 receptor-mediated free water retention.

Lastly, while AVP-induced platelet activation via V1a receptor may theoretically enhance thrombotic risk, clinical studies across different shock states have not documented an increased incidence of thromboembolic events in patients receiving AVP.^19,20^

## Rationale of vasopressin use in cardiogenic shock

Vasopressin is a drug primarily used in septic shock, a form of distributive shock characterized by vasodilation and low SVR. In this context, the Surviving Sepsis Campaign guidelines recommend it as a second-line vasopressor, to be considered after norepinephrine.^11^

In patients with cardiogenic shock, the use of AVP requires careful clinical judgment. European and American guidelines do not assign a defined role to vasopressin in this setting, largely due to the lack of robust supporting evidence. Its ability to raise SVR and MAP without stimulating β-adrenergic receptors—and thereby avoiding a direct increase in myocardial oxygen consumption—may offer theoretical advantages. However, these benefits must be weighed against the risk of increased afterload, which may further impair an already failing left ventricle. The potential for worsening myocardial ischaemia, particularly in patients with compromised coronary perfusion, should not be underestimated. The primary therapeutic goal remains to restore systemic perfusion without imposing additional strain on the injured myocardium. In this setting, haemodynamic monitoring is essential—either invasively with a pulmonary artery catheter or noninvasively with echocardiography—to assess cardiac output and vascular resistance and titrate vasoactive therapy.

From a pathophysiological perspective, patients with various forms of shock often display a biphasic pattern in vasopressin secretion. In the early phases, AVP levels typically rise as part of a compensatory response aimed at preserving arterial pressure and fluid homeostasis. However, as shock progresses, plasma AVP concentrations may become inappropriately low relative to the severity of hypotension, a condition referred to as ‘relative vasopressin deficiency’. This deficiency is thought to play a key role in the persistence of hypotension that proves resistant to standard vasopressor therapy.^21^

Several mechanisms may underlie this phenomenon, including depletion of AVP stores in the posterior pituitary and impaired function of osmoregulatory or baroreceptor pathways. When this relative deficiency is compounded by the well-documented catecholamine hyporesponsiveness observed in late-stage shock, the administration of exogenous AVP becomes a rational and potentially life-saving therapeutic option.

Refractory hypotension can also arise in advanced stages of cardiogenic shock, not only in distributive forms. The downward spiral of cardiogenic shock, where reduced cardiac output leads to tissue hypoperfusion, hypoxia, and acidosis, triggers the release of numerous inflammatory mediators that disrupt vascular tone and peripheral resistance. Among these, biomarkers such as adrenomedullin and angiopoietin-2 are emerging as indicators of endothelial dysfunction and inappropriate vasodilation,^22,23^ with potential diagnostic, prognostic, and therapeutic relevance.^24^ Measurement of circulating copeptin, which reflects endogenous AVP release,^25^ although not yet routine in clinical practice, could help identify patients with relative vasopressin deficiency.^26^ Copeptin levels also appear to carry independent prognostic significance in cardiogenic shock.^27^ Accordingly, elevated adrenomedullin and angiopoietin-2 or inappropriately low copeptin may help flag candidates for AVP therapy and, subsequently, guide vasopressor weaning alongside clinical and haemodynamic parameters.

## Clinical evidence on vasopressin use in shock

The most robust clinical evidence supporting the use of AVP comes from randomized controlled trials in patients with septic shock. In the VASST (Vasopressin and Septic Shock Trial), the addition of low-dose AVP (up to 0.03 U/min) to norepinephrine did not improve overall survival compared to norepinephrine alone. Notably, no increase in adverse events was observed, although the study excluded patients with acute coronary syndromes or advanced heart failure.^13^

Similarly, the VANISH (Vasopressin vs Noradrenaline as Initial Therapy in Septic Shock) trial evaluated the early use of AVP in septic shock and found no significant improvement in renal outcomes or mortality. However, patients in the AVP group demonstrated a lower requirement for renal replacement therapy.^28^ The theoretical renal advantage of vasopressin lies in its preferential vasoconstriction of the efferent arteriole, which helps preserve glomerular filtration pressure. In contrast, norepinephrine induces vasoconstriction in both afferent and efferent arterioles, which may compromise glomerular perfusion and contribute to acute kidney injury.^29^

In the VANCS (Vasopressin vs. Norepinephrine in Vasoplegic Shock after Cardiac Surgery) trial, AVP compared with norepinephrine in vasoplegic shock after cardiac surgery was associated with lower rates of acute kidney injury, shorter ICU stay, and reduced incidence of atrial fibrillation, without differences in ischaemic or myocardial infarction events.^30^

With regard to cardiogenic shock, evidence supporting vasopressin use remains scarce and largely limited to retrospective studies (see *Table 1*). One such study found that the incidence of adverse events related to vasopressin was proportional to the duration of infusion. Approximately 55% of patients were pressure responders to AVP, although this was not associated with 30-day mortality, and 56% of patients were already on ExtraCorporeal Membrane Oxygenation (ECMO) support.^14^

**Table 1:** Evidence on vasopressin use in cardiogenic shock

Study	Design and population	Main findings	Limitations
Nguyen *et al*., 2023^14^	Retrospective cohort; patients with CS, 56% on ECMO. *n* = 100	55% were AVP pressure responders; response not associated with 30-day mortality	High ECMO rate; no mortality benefit shown
Beyls *et al*., 2025^31^	Retrospective cohort with historical controls; CS ≥ SCAI stage C. *n* = 59 in the AVP group	No significant differences in 90-day mortality or serious adverse events	>50% were post-cardiac surgery; not representative of classical CS
Sarma *et al*., 2025^32^	Retrospective cohort; excluded post-operative and ECMO patients. *n* = 207 in the AVP group	Benefit in high-dose vasopressor subgroup (NE > 0.3 µg/kg/min); no benefit by phenotype	Retrospective; lack of invasive haemodynamic data

AVP, arginine vasopressin; CS, cardiogenic shock; ECMO, extracorporeal membrane oxygenation; NE, norepinephrine; SCAI, Society for Cardiovascular Angiography and Interventions.

Another retrospective cohort study comparing patients with cardiogenic shock (Society for Cardiovascular Angiography and Interventions [SCAI] stage C or higher) who received AVP to a historical control group not treated with AVP found no significant differences in 90-day mortality or serious adverse events.^31^ It should be noted, however, that more than 50% of the patients in this study had post-cardiac surgery shock.

The most compelling evidence supporting vasopressin use in cardiogenic shock comes from a recent retrospective cohort study conducted at the Mayo Clinic. This large analysis included patients with cardiogenic shock requiring vasopressor support, excluding those post-cardiac surgery or receiving ECMO. The greatest in-hospital mortality benefit was observed among patients requiring high-dose vasopressors (norepinephrine equivalent > 0.3 µg/kg/min) who received adjunctive AVP.^32^ No specific shock aetiology was associated with greater benefit, and invasive haemodynamic data were not available. Three cardiogenic shock phenotypes—non-congested, cardiorenal, and cardiometabolic—were analysed, as defined by a recent phenotyping study.^33^ AVP use was not associated with mortality benefit in any subgroup. Notably, it was more frequently administered in the cardiometabolic phenotype, which also showed the highest mortality (used in 33% of these patients, with a mortality rate of 59%).

These findings suggest that in advanced cardiogenic shock—particularly in cases requiring high vasopressor doses, indicative of low SVR and a possible mixed shock state—vasopressin may offer some benefit, either by augmenting perfusion pressure or allowing catecholamine dose reduction. However, the current evidence remains very limited.

## Cardiogenic shock phenotypes

**Figure 1:**
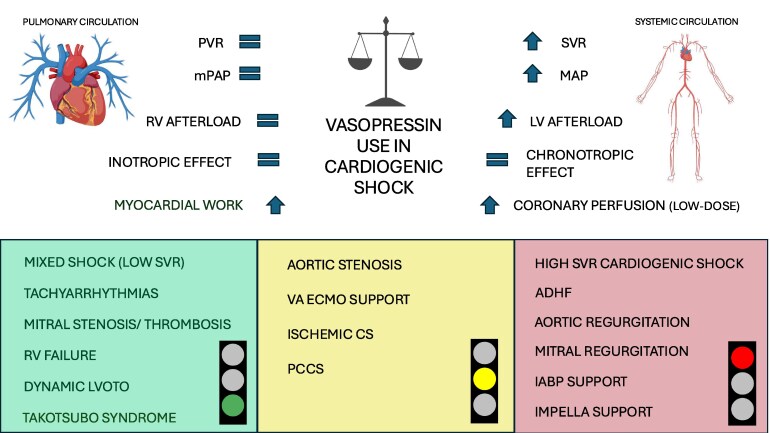
(Graphical Abstract) Potential haemodynamic effects and clinical scenarios for vasopressin use in cardiogenic shock. The diagram summarizes the pharmacodynamic profile of vasopressin, highlighting its effects on SVR, PVR, MAP, mPAP, myocardial work, and coronary perfusion, as well as its neutral impact on inotropy and chronotropy. Clinical phenotypes where vasopressin may be beneficial are shown in green (on the left—e.g. mixed shock with low SVR, tachyarrhythmias, right ventricular failure, dynamic LV outflow tract obstruction, mitral stenosis/thrombosis, and Takotsubo syndrome). Conversely, scenarios in which vasopressin may be detrimental are shown in red (on the right—e.g. aortic and mitral regurgitation, cardiogenic shock with high SVR, and mechanical circulatory support with IABP or Impella). In yellow scenarios, in which its use must be carefully evaluated (in the middle). ADHF, acute decompensated heart failure; CS, cardiogenic shock; IABP, Intra-aortic balloon pump; LV, left ventricular; left ventricular outflow tract obstruction; MAP, mean arterial pressure; MCS,(mechanical circulatory support; mPAP, mean pulmonary artery pressure; PCCS, post cardiotomy cardiogenic shock; PVR, pulmonary vascular resistance; RV, right ventricular; SVR, systemic vascular resistance; VA ECMO, veno-arterial extracorporeal membrane oxygenation. Created with BioRender

**Figure 2:**
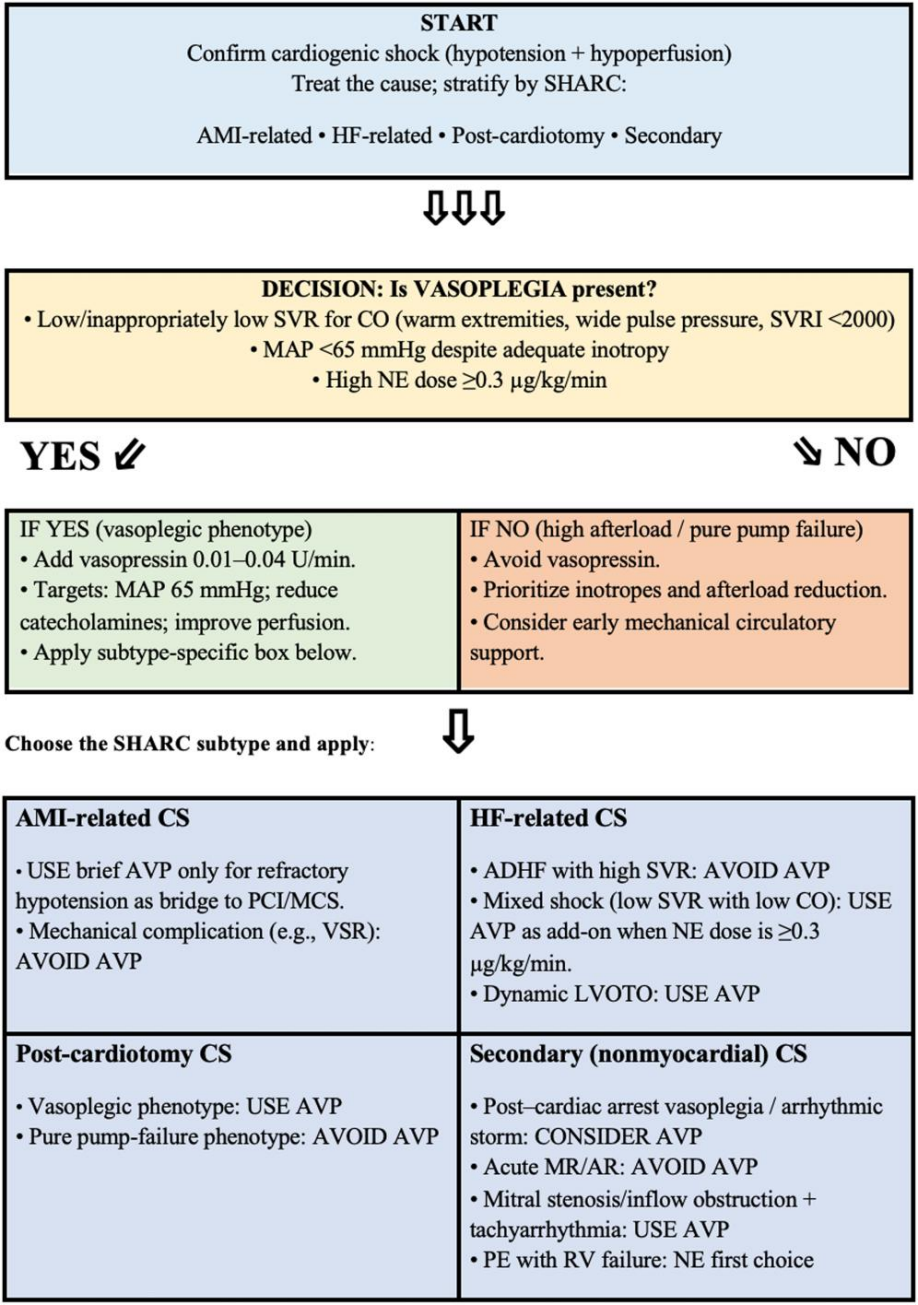
Vasopressin algorithm for cardiogenic shock (SHARC-based). Abbreviations: ADHF, acute decompensated heart failure; AMI, acute myocardial infarction; AR, aortic regurgitation; AVP, vasopressin; CO, cardiac output; LVOTO, left ventricular outflow tract obstruction; MCS; mechanical circulatory support; MR, mitral regurgitation; NE, norepinephrine; PCI, percutaneous coronary intervention; PE, pulmonary embolism; RV, right ventricle; SVR, systemic vascular resistance; SVRI, systemic vascular resistance index; VSR, ventricular septal rupture

With this pathophysiological and haemodynamic framework established, we now move to specific clinical phenotypes of cardiogenic shock in which vasopressin may or may not be appropriate (*see Figure 1*), starting from the classification proposed by the Shock Academic Research Consortium (SHARC)^34^ (see *Figure 2* for a clinical algorithm).

### AMI—related cardiogenic shock

This group includes cardiogenic shock caused by acute myocardial infarction (AMI), resulting from severe contractile dysfunction due to acute ischaemic injury, mechanical complications of the infarction, or infarction-related arrhythmias. Typically, the abrupt loss of pump function prevents the development of adequate compensatory mechanisms. These patients usually present with hypotension, pulmonary congestion, and systemic hypoperfusion, requiring urgent stabilization to maintain vital organ perfusion and allow time for causal therapy. In some cases, mechanical circulatory support (MCS) becomes the only viable option. Vasopressin is generally not recommended in this setting, particularly in patients with pulmonary oedema or respiratory failure, where effective left ventricular unloading is essential. However, it may be considered as a temporary strategy in cases of refractory hypotension, serving as a bridge to MCS or definitive resolution of the underlying cause, such as acute coronary thrombosis.

Occasionally, cardiogenic shock may arise several days after the initial ischaemic event, due to mechanical complications in untreated or late-presenting myocardial infarction, such as ventricular septal rupture (VSR). In this scenario, vasopressin should be avoided, as its pure vasoconstrictive action would increase SVR and worsen the left-to-right shunt. Mechanical support with intra-aortic balloon pump (IABP) and vasodilator therapy is generally preferred.^35^ If a vasopressor is deemed necessary to maintain adequate MAP, norepinephrine should be favoured due to its concomitant positive inotropic effects.

*Key clinical message*: In AMI-related cardiogenic shock, prioritize rapid cause resolution and early MCS with a left-sided pVAD for LV unloading. Avoid AVP in mechanical complications, such as VSR or papillary muscle rupture.

### Heart failure—related cardiogenic shock

This broad category includes patients with cardiogenic shock caused by primary myocardial dysfunction not due to an ongoing myocardial infarction.^34^ Given its heterogeneity, we will examine several distinct subtypes.

#### ADHF phenotype

Vasopressin is generally not the agent of choice in cardiogenic shock due to acute decompensated heart failure (ADHF) with elevated SVR (typically SVRI > 2200 dyn·s·cm^−5^·m^2^). In such cases, therapy should prioritize inotropic support and vasodilators. Agents like nitroprusside may improve ventriculo-arterial coupling and enhance forward flow without significantly reducing systemic blood pressure.^36,37^ This approach is especially suitable in normotensive patients with evidence of hypoperfusion, a scenario where vasopressin is usually not indicated.

*Key clinical message*: AVP must be avoided in patients with low CO and high SVR.

#### Mixed shock phenotype

The condition of mixed shock, in which initially cardiogenic shock evolves into a distributive-inflammatory form, occurs in approximately 25% of patients with cardiogenic shock and is associated with significantly higher in-hospital mortality (53% vs. 28% in pure cardiogenic shock, according to the cited cohort).^38^ The haemodynamic profile of this ‘warm and wet’ shock is characterized by low cardiac output, elevated filling pressures, and low SVR—or SVR that is disproportionately low relative to the degree of cardiac output and inadequate to sustain an appropriate MAP.^39^

This condition may arise as a progression of cardiogenic shock itself, due to persistent tissue hypoperfusion, metabolic acidosis, and the subsequent release of inflammatory mediators, or from the frequent development of concomitant infections in patients with multiple invasive devices.

A clinical scenario that can mimic this phenotype is sepsis-induced cardiomyopathy, where ventricular dysfunction is primarily driven by a cytokine storm in the context of sepsis and may present with a similar haemodynamic pattern.^40,41^

These clinical scenarios share the combination of low cardiac output and low SVR. In this setting, vasopressin may be beneficial, particularly when catecholamines are ineffective or should be minimized. Notably, the greatest benefit from AVP has been observed in cases requiring high-dose vasopressor support.^32^

*Key clinical message*: AVP may be added to norepinephrine in mixed cardiogenic shock when high NE doses are required (≥0.3 µg/kg/min), although the evidence is limited.

#### Obstructive phenotype

Another clinical setting where vasopressin may prove particularly valuable is cardiogenic shock associated with dynamic left ventricular outflow tract obstruction (LVOTO).

A classic example is the so-called ‘suicide left ventricle’ occurring after aortic valve replacement. In this scenario, the sudden drop in left ventricular afterload can precipitate dynamic outflow tract obstruction, especially in hypertrophied ventricles. Management typically includes beta-blockade and volume expansion to increase preload, but restoring afterload through pure vasopressors, such as vasopressin or phenylephrine, is often essential to resolve the post-valve-replacement pressure mismatch and restore haemodynamic stability.^42^

Similarly, patients with hypertrophic obstructive cardiomyopathy (HOCM) and severe hypotension—particularly in the setting of tachyarrhythmias—may benefit from vasopressin administration.^43^ The benefit is even more pronounced in neonates with HOCM and haemodynamic instability, as vasopressin does not increase pulmonary vascular resistance, which is already elevated in this population^44^

Finally, in septic shock patients with LVOTO due to hypercontractility, vasopressin has been reported to help reduce norepinephrine requirements and achieve improved haemodynamic and respiratory stability.^45^

In these clinical scenarios, a pure alpha-agonist such as phenylephrine may also represent a valid alternative.^46^

*Key clinical message*: In cardiogenic shock due to dynamic LVOTO, vasopressin or phenylephrine should be considered the vasopressors of choice.

#### Takotsubo syndrome

Vasopressin may also be useful in cases of Takotsubo syndrome complicated by LVOTO and cardiogenic shock. In these patients, adrenergic agonists and inotropes are typically contraindicated, as they can exacerbate the obstruction due to basal hypercontractility. First-line treatment consists of intravenous beta-blockers and cautious fluid administration, though care must be taken when systolic anterior motion of the mitral valve and severe mitral regurgitation are present.^47^ In this setting, AVP can be crucial for haemodynamic stabilization, as it increases vascular tone without worsening dynamic obstruction.

An additional therapeutic strategy to limit sympathetic activation without adversely affecting haemodynamics may include neuromodulation through left stellate ganglion blockade.^48^ Sometimes left-sided p-VAD (e.g. Impella device) placement is necessary to overcome obstruction and facilitate LV unloading.^49,50^

*Key clinical message*: In TTS complicated by LVOTO and cardiogenic shock, consider AVP as first line vasopressor, along with short-acting beta blockers. Consider MCS with left-sided p-VAD.

### Post—cardiotomy cardiogenic shock

Postcardiotomy cardiogenic shock (PCCS) is a low–cardiac-output state driven by varying contributions of myocardial stunning, ischaemia–reperfusion injury, and the cardiopulmonary-bypass-related systemic inflammatory response, often causing profound vasoplegia that compounds pump failure.^51^ These mechanisms overlay pre-existing ischaemic or valvular disease; elevated preoperative hs-troponin and NT-proBNP help identify higher-risk patients.^52^ When vasoplegia predominates, AVP is physiologically attractive: V1a-mediated vasoconstriction restores vascular tone, enabling catecholamine-sparing. Its neutral effect on pulmonary vascular resistance is advantageous when right ventricular function is impaired and inhaled vasodilators are used for severe pulmonary hypertension. In postoperative vasoplegia, AVP vs norepinephrine reduced atrial fibrillation, acute kidney injury, and ICU stay in the VANCS trial, which enrolled patients with a preserved cardiac index.^30^ Nevertheless, high-quality evidence in PCCS remains limited; AVP can be deployed in a vasoplegic-predominant phenotype alongside optimized loading conditions and timely MCS. Further prospective studies are needed to better clarify its role in PCCS.

*Key clinical message*: In PCCS, consider AVP when vasoplegia predominates or with right-ventricular dysfunction, while simultaneously evaluating for early MCS. Avoid AVP in isolated left-sided pump failure.

### Nonmyocardial (secondary) cardiogenic shock

According to the SHARC classification, this category includes conditions in which pump failure is secondary to non-myocardial problems—not primary contractile depression—such as severe arrhythmias, acute valvular lesions, pericardial tamponade, and pulmonary vascular obstruction.^34^ Each has distinct haemodynamic targets and implications for vasopressor choice.

#### Arrhythmic phenotype

During cardiac arrest, international guidelines recommend epinephrine as the sole vasopressor for both shockable and non-shockable rhythms.^53,54^ Meta-analyses have shown no benefit for vasopressin alone or added to epinephrine,^55,56^ so its use is not recommended in this setting.

Conversely, AVP may play a valuable role in the management of post-cardiac arrest syndrome. Following resuscitation, particularly after prolonged no-flow or low-flow states, patients often develop a systemic inflammatory response and vasodilation triggered by ischaemia-reperfusion injury, metabolic acidosis, and the accumulation of catabolites.^57^ This is frequently compounded by myocardial injury leading to impaired contractility, neurological damage with autonomic dysfunction and central fevers, and infectious complications, such as aspiration pneumonia or device-related infections. In this context, AVP can help restore haemodynamic stability, especially when catecholamines are limited by the risk of recurrent arrhythmias.

AVP may also be beneficial in patients experiencing haemodynamic instability due to tachyarrhythmias, both ventricular and supraventricular. In electrical storm from recurrent ventricular tachycardia, management typically aims to reduce adrenergic drive through intravenous antiarrhythmics, neuromodulation (e.g. stellate ganglion block), and deep sedation. Since sedative agents, beta-blockers, and other antiarrhythmics like amiodarone frequently cause hypotension, vasopressor support may become necessary. In such cases, AVP offers a non-adrenergic mechanism of action and is therefore suitable.

The benefits of vasopressin become even more relevant in the presence of predominant right ventricular failure during tachyarrhythmias, given its neutral effect on pulmonary vascular resistance.^8^

*Key clinical message*: Do not use AVP during cardiac arrest. Consider AVP in post-cardiac arrest syndrome. Consider AVP in haemodynamic instability due to ongoing or recurrent tachyarrhythmias.

#### Acute valvular disease

In cardiogenic shock due to severe valvular disease, vasopressin may have a role in selected circumstances. Its use is generally not appropriate when afterload reduction is beneficial, such as in acute severe mitral or aortic regurgitation.

A different scenario applies to mitral stenosis, where heart rate control and diastolic filling time are critical to preserving cardiac output. Acute decompensation frequently arises in the context of supraventricular tachyarrhythmias, most often atrial fibrillation. In patients requiring vasopressor support to maintain adequate MAP, vasopressin or phenylephrine may be preferred because of their neutral chronotropic effect.^46,58^ A similar rationale applies to cardiogenic shock caused by obstruction to left ventricular inflow, such as from atrial myxomas, intracardiac masses, or acute thrombosis of a mechanical mitral prosthesis. In these obstructive shock scenarios, left ventricular systolic function is usually preserved and not impaired by increased afterload, making vasopressors more appropriate than inotropes as a bridge to definitive intervention.

In severe aortic stenosis, cardiogenic shock usually follows an acute trigger, such as ischaemia, tachyarrhythmia, sepsis, or acute kidney injury. Left ventricular afterload is determined by the fixed valvular obstruction rather than SVR. With hypotension, preserving coronary perfusion becomes essential to prevent a downward spiral of worsening ischaemia and declining ventricular performance. Pressors should restore perfusion without adding chronotropy; therefore, vasopressin or phenylephrine are preferred, particularly in the presence of tachyarrhythmias.^46,58^ Norepinephrine is reasonable when some inotropy is desired, given its mild β-adrenergic effect. In patients with tachyarrhythmias, vasopressin can be combined with short-acting intravenous β-blockers to achieve haemodynamic stability as a bridge to intervention (e.g. balloon aortic valvuloplasty).

*Key clinical message*: Avoid AVP in cardiogenic shock from acute severe mitral or aortic regurgitation. Consider AVP in severe mitral stenosis, LV inflow obstruction, or severe aortic stenosis.

#### Pericardial tamponade

Pericardial tamponade is a haemodynamic emergency in which rising intrapericardial pressure impairs diastolic filling and rapidly depresses cardiac output. Definitive therapy is urgent pericardial drainage. While preparing for pericardiocentesis, brief stabilization includes fluids and a vasopressor; norepinephrine may be preferred for its α-mediated venous constriction that augments venous return, while rising MAP.^59^

#### Acute pulmonary embolism

There is no clinical evidence supporting vasopressin in shock due to pulmonary embolism (PE), despite a plausible pharmacologic rationale. Although robust adult data are lacking, low-dose AVP may be a reasonable vasopressor in acute RV failure, as it does not worsen right ventricular–pulmonary arterial coupling.^60^ However, norepinephrine remains first-line to support MAP and preload in acute PE.

*Key clinical message*: Despite a pharmacologic rationale, there is no clinical evidence for AVP in acute PE-related shock; norepinephrine is the first-line vasopressor.

## Vasopressin and MCS

MCS is often required in cases of worsening or refractory cardiogenic shock, and the haemodynamic effects of these devices warrant dedicated consideration.

IABP provides ventricular support primarily by reducing left ventricular afterload. Its augmentation of diastolic pressure improves coronary and cerebral perfusion.^61^ Pure vasoconstrictors such as vasopressin increase afterload and counteract IABP’s core benefit, so they should be avoided whenever possible, especially in ADHF and in severe mitral or aortic regurgitation.

A similar principle applies to Impella support (left-sided pVAD). Impella directly unloads the left ventricle and significantly increases cardiac output (with the Impella CP delivering up to 3.5–4.0 L/min at peak performance). In a retrospective single-centre cohort of Impella-supported shock (2.5, CP, and 5.0), regimens relying solely on vasopressors were associated with higher mortality than those including an inotrope; vasopressin use was associated with an hazard ratio of approximately 2.7 for mortality. All vasopressors studied—norepinephrine, vasopressin, dopamine, epinephrine, and phenylephrine—were significantly associated with increased mortality, in contrast to dobutamine and milrinone, which were not.^62^ Although confounding by severity is likely, this supports using the lowest effective vasopressor dose and prioritizing inotropy and afterload optimization when feasible in left-sided pVAD patients.

Veno-arterial extracorporeal membrane oxygenation (VA-ECMO) often necessitates the use of vasopressors to maintain adequate MAP. ECMO is frequently deployed in cases of advanced cardiogenic shock or refractory cardiac arrest, and systemic inflammatory response is common in these clinical scenarios. Moreover, the passage of blood through the non-endothelialized ECMO circuit can trigger widespread activation of the innate immune system, resulting in cytokine release, inflammation, and systemic vasodilation,^63^ along with coagulation dysregulation. In this context, vasopressin may be beneficial, as a catecholamine-sparing agent. In an animal model of refractory cardiac arrest treated after 30 min of resuscitation with VA-ECMO initiation, vasopressin administration was associated with faster lactate clearance, reduced fluid requirements, and lower incidence of pulmonary oedema compared to norepinephrine.^64^ However, because vasopressors further exacerbate LV afterload, early implementation of LV unloading strategies should be considered.

*Key clinical message*: AVP should be avoided in patients supported with IABP or left-sided Impella, while it may be considered in VA-ECMO with vasoplegia.

## Future directions

Ideal evidence would come from randomized controlled trials testing AVP across cardiogenic-shock phenotypes, but patient instability and heterogeneity make such studies challenging.

Priority should be given to prospective, pragmatic, biomarker- and phenotype-enriched studies. Enrolment should target mixed shock with low cardiac index and inappropriately low SVR, postcardiotomy shock with a prominent vasoplegic component, severe aortic stenosis with hypotension, and shock from right ventricular failure. Stratification by circulating biomarkers—adrenomedullin, angiopoietin-2, and copeptin—together with invasive or echocardiographic haemodynamics should guide inclusion and response assessment. Registry-embedded randomization comparing norepinephrine alone vs norepinephrine + AVP vs AVP-first strategy, with protocolized dosing and weaning, is feasible. Key endpoints should include days alive and free of organ support, acute kidney injury, and need for renal replacement therapy, clinically significant arrhythmias, ICU length of stay, and 30-day mortality. Prespecified analyses in patients on mechanical circulatory support are essential.

## Conclusions

A pathophysiological approach is essential to identify the clinical contexts of cardiogenic shock in which vasopressin may be beneficial and others where it may be potentially harmful.

Although the available evidence is very limited and largely derived from retrospective studies, clinical settings such as mixed or advanced cardiogenic shock with low SVR, tachyarrhythmias, right ventricular failure, Takotsubo syndrome, severe aortic stenosis, mitral inflow obstruction, and dynamic outflow tract obstruction are scenarios in which vasopressin may play a valuable role.

## References

[1] Del Buono MG, La Vecchia G, D'Aiello A, Pedicino D, Pinnacchio G, Genuardi L, et al Clinical characteristics, management, and outcomes in cardiogenic shock: insights from a high-volume Italian cardiac intensive care unit. J Cardiovasc Pharmacol 2024;84:210–9. 10.1097/FJC.0000000000001584 39115720 PMC11309343

[2] Lüsebrink E, Binzenhöfer L, Adamo M, Lorusso R, Mebazaa A, Morrow DA, et al Cardiogenic shock. Lancet 2024;404:2006–20. 10.1016/S0140-6736(24)01818-X 39550175

[3] McDonagh TA, Metra M, Adamo M, Gardner RS, Baumbach A, Böhm M, et al 2021 ESC Guidelines for the diagnosis and treatment of acute and chronic heart failure. Eur Heart J 2021;42:3599–726. 10.1093/eurheartj/ehab368 Erratum in: Eur Heart J 2021;42:4901. 10.1093/eurheartj/ehab670. 34447992

[4] Maybauer MO, Maybauer DM, Enkhbaatar P, Traber DL. Physiology of the vasopressin receptors. Best Pract Res Clin Anaesthesiol 2008;22:253–63. 10.1016/j.bpa.2008.03.003 18683472

[5] Mizota T, Fujiwara K, Hamada M, Matsukawa S, Segawa H. Effect of arginine vasopressin on systemic and pulmonary arterial pressure in a patient with pulmonary hypertension secondary to pulmonary emphysema: a case report. JA Clin Rep 2017;3:1. 10.1186/s40981-016-0072-3 29492440 PMC5813718

[6] Korkames G, Brink H, Peitz G. Effect of norepinephrine versus vasopressin on pulmonary artery pressure. Crit Care Med 2022;50:546. 10.1097/01.ccm.0000810708.23191.69

[7] Evora PR, Pearson PJ, Schaff HV. Arginine vasopressin induces endothelium-dependent vasodilatation of the pulmonary artery: V1-receptor-mediated production of nitric oxide. Chest 1993;103:1241–5. 10.1378/chest.103.4.1241 8131474

[8] Wallace AW, Tunin CM, Shoukas AA. Effects of vasopressin on pulmonary and systemic vascular mechanics. Am J Physiol 1989;257:H1228–34. 10.1152/ajpheart.1989.257.4.H1228 2801982

[9] Jeon Y, Ryu JH, Lim YJ, Kim CS, Bahk JH, Yoon SZ, et al Comparative hemodynamic effects of vasopressin and norepinephrine after milrinone-induced hypotension in off-pump coronary artery bypass surgical patients. Eur J Cardiothorac Surg 2006;29:952–6. 10.1016/j.ejcts.2006.02.032 16675238

[10] Jakowenko ND, Murata J, Kopp BJ, Erstad BL. Influence of timing and catecholamine requirements on vasopressin responsiveness in critically ill patients with septic shock. J Intensive Care Med 2022;37:1512–9. 10.1177/08850666221081836 35195486

[11] Evans L, Rhodes A, Alhazzani W, Antonelli M, Coopersmith CM, French C, et al Surviving sepsis campaign: international guidelines for management of sepsis and septic shock 2021. Intensive Care Med 2021;47:1181–247. 10.1007/s00134-021-06506-y 34599691 PMC8486643

[12] Meyfroidt G, Gunst J, Martin-Loeches I, Smith M, Robba C, Taccone FS, et al Management of the brain-dead donor in the ICU: general and specific therapy to improve transplantable organ quality. Intensive Care Med 2019;45:343–53. 10.1007/s00134-019-05551-y 30741327 PMC7095373

[13] Russell JA, Walley KR, Singer J, Gordon AC, Hébert PC, Cooper DJ, et al Vasopressin versus norepinephrine infusion in patients with septic shock. N Engl J Med 2008;358:877–87. 10.1056/NEJMoa067373 18305265

[14] Nguyen M, Berthoud V, Rizk A, Bouhemad B, Guinot PG. Real life use of vasopressin in patients with cardiogenic shock: a retrospective cohort analysis. Crit Care 2023;27:291. 10.1186/s13054-023-04574-8 37468928 PMC10357707

[15] Jolly S, Newton G, Horlick E, Seidelin PH, Ross HJ, Husain M, et al Effect of vasopressin on hemodynamics in patients with refractory cardiogenic shock complicating acute myocardial infarction. Am J Cardiol 2005;96:1617–20. 10.1016/j.amjcard.2005.07.078 16360345

[16] Overgaard CB, Dzavík V. Inotropes and vasopressors: review of physiology and clinical use in cardiovascular disease. Circulation 2008;118:1047–56. 10.1161/CIRCULATIONAHA.107.728840 18765387

[17] Asfar P, Radermacher P. Vasopressin and ischaemic heart disease: more than coronary vasoconstriction? Crit Care 2009;13:169. 10.1186/cc7954 19664189 PMC2750154

[18] McIntyre WF, Um KJ, Alhazzani W, Lengyel AP, Hajjar L, Gordon AC, et al Association of vasopressin plus catecholamine vasopressors vs catecholamines alone with atrial fibrillation in patients with distributive shock: a systematic review and meta-analysis. JAMA 2018;319:1889–900. 10.1001/jama.2018.4528 29801010 PMC6583502

[19] Doepker BA, Lucarelli MR, Lehman A, Shirk MB. Thromboembolic events during continuous vasopressin infusions: a retrospective evaluation. Ann Pharmacother 2007;41:1383–9. 10.1345/aph.1H498 17684034

[20] Sims CA, Holena D, Kim P, Pascual J, Smith B, Martin N, et al Effect of low-dose supplementation of arginine vasopressin on need for blood product transfusions in patients with trauma and hemorrhagic shock: a randomized clinical trial. JAMA Surg 2019;154:994–1003. 10.1001/jamasurg.2019.2884 31461138 PMC6714462

[21] Landry DW, Levin HR, Gallant EM, Ashton RC Jr, Seo S, D'Alessandro D, et al Vasopressin deficiency contributes to the vasodilation of septic shock. Circulation 1997;95:1122–5. 10.1161/01.cir.95.5.1122 9054839

[22] Link A, Pöss J, Rbah R, Barth C, Feth L, Selejan S, et al Circulating angiopoietins and cardiovascular mortality in cardiogenic shock. Eur Heart J 2013;34:1651–62. 10.1093/eurheartj/ehs487 23349297

[23] Tolppanen H, Rivas-Lasarte M, Lassus J, Sans-Roselló J, Hartmann O, Lindholm M, et al Adrenomedullin: a marker of impaired hemodynamics, organ dysfunction, and poor prognosis in cardiogenic shock. Ann Intensive Care 2017;7:6. 10.1186/s13613-016-0229-2 28050899 PMC5209311

[24] Jozwiak M, Lim SY, Si X, Monnet X. Biomarkers in cardiogenic shock: old pals, new friends. Ann Intensive Care 2024;14:157. 10.1186/s13613-024-01388-x 39414666 PMC11485002

[25] Jochberger S, Dörler J, Luckner G, Mayr VD, Wenzel V, Ulmer H, et al The vasopressin and copeptin response to infection, severe sepsis, and septic shock. Crit Care Med 2009;37:476–82. 10.1097/CCM.0b013e3181957532 19114902

[26] Jeon K, Song JU, Chung CR, Yang JH, Suh GY. Incidence of hypotension according to the discontinuation order of vasopressors in the management of septic shock: a prospective randomized trial (DOVSS). Crit Care 2018;22:131. 10.1186/s13054-018-2034-9 29784057 PMC5961479

[27] Meyer B, Wexberg P, Struck J, Bergmann A, Morgenthaler N, Heinz G, et al Copeptin is a strong and independent predictor of outcome in cardiogenic shock. Crit Care 2009;13:P383. 10.1186/cc7547

[28] Gordon AC, Mason AJ, Thirunavukkarasu N, Perkins GD, Cecconi M, Cepkova M, et al Effect of early vasopressin vs norepinephrine on kidney failure in patients with septic shock: the VANISH randomized clinical trial. JAMA 2016;316:509–18. 10.1001/jama.2016.10485 27483065

[29] Edwards RM, Trizna W, Kinter LB. Renal microvascular effects of vasopressin and vasopressin antagonists. Am J Physiol 1989;256:F274–8. 10.1152/ajprenal.1989.256.2.F274 2916660

[30] Hajjar LA, Vincent JL, Galas FRBG, Rhodes A, Landoni G, Osawa EA, et al Vasopressin versus norepinephrine in patients with vasoplegic shock after cardiac surgery: the VANCS randomized controlled trial. Anesthesiology 2017;126:85–93. 10.1097/ALN.0000000000001434 27841822

[31] Beyls C, Hanquiez T, Mollet N, Sarfati Y, Zerima A, Chafiki S, et al The effect of vasopressin on 90-day mortality in patients with cardiogenic shock: a retrospective cohort study using propensity score-weighted analysis. Cardiovasc Ther 2025;2025:9920490. 10.1155/cdr/9920490 40469854 PMC12136860

[32] Sarma D, Smith R, Padkins M, Rali AS, Vallabhajosyula S, Khanna AK, et al Association between vasopressin administration and mortality in patients with cardiogenic shock. Am Heart J 2025;286:88–96. 10.1016/j.ahj.2025.03.009 40120706

[33] Zweck E, Thayer KL, Helgestad OKL, Kanwar M, Ayouty M, Garan AR, et al Phenotyping cardiogenic shock. J Am Heart Assoc 2021;10:e020085. 10.1161/JAHA.120.020085 34227396 PMC8483502

[34] Waksman R, Pahuja M, van Diepen S, Proudfoot AG, Morrow D, Spitzer E, et al Standardized definitions for cardiogenic shock research and mechanical circulatory support devices: scientific expert panel from the Shock Academic Research Consortium (SHARC). Circulation 2023;148:1113–26. 10.1161/CIRCULATIONAHA.123.064527 37782695 PMC11025346

[35] Schlotter F, Huber K, Hassager C, Halvorsen S, Vranckx P, Pöss J, et al Ventricular septal defect complicating acute myocardial infarction: diagnosis and management. A clinical consensus statement of the Association for Acute CardioVascular Care (ACVC) of the ESC, the European Association of Percutaneous Cardiovascular Interventions (EAPCI) of the ESC and the ESC Working Group on Cardiovascular Surgery. Eur Heart J 2024;45:2478–92. 10.1093/eurheartj/ehae363 38888906

[36] Mullens W, Abrahams Z, Francis GS, Skouri HN, Starling RC, Young JB, et al Sodium nitroprusside for advanced low-output heart failure. J Am Coll Cardiol 2008;52:200–7. 10.1016/j.jacc.2008.02.083 18617068

[37] Garatti L, Frea S, Bocchino PP, Angelini F, Cingolani M, Sacco A, et al Sodium nitroprusside in acute heart failure: a multicenter historic cohort study. Int J Cardiol 2022;369:37–44. 10.1016/j.ijcard.2022.08.009 35944767 PMC9771588

[38] Baldetti L, Gallone G, Filiberti G, Pescarmona L, Cesari A, Rizza V, et al Mixed shock complicating cardiogenic shock: frequency, predictors, and clinical outcomes. Circ Heart Fail 2024;17:e011404. 10.1161/CIRCHEARTFAILURE.123.011404 38979611

[39] van Diepen S, Pöss J, Senaratne JM, Gage A, Morrow DA. Mixed cardiogenic shock: a proposal for standardized classification, a hemodynamic definition, and framework for management. Circulation 2024;150:1459–68. 10.1161/CIRCULATIONAHA.124.069508 39466889

[40] L’Heureux M, Sternberg M, Brath L, Turlington J, Kashiouris MG. Sepsis-induced cardiomyopathy: a comprehensive review. Curr Cardiol Rep 2020;22:35. 10.1007/s11886-020-01277-2 32377972 PMC7222131

[41] Aissaoui N, Boissier F, Chew M, Singer M, Vignon P. Sepsis-induced cardiomyopathy. Eur Heart J 2025;46:3339–53. 10.1093/eurheartj/ehaf340 40439150

[42] Koliastasis L, Drakopoulou M, Latsios G, Apostolos A, Ktenopoulos N, Katsaros O, et al Overcoming the obstacle of suicide left ventricle after transcatheter aortic valve replacement phenomenon. JACC Case Rep 2023;26:102065. 10.1016/j.jaccas.2023.102065 38094179 PMC10715948

[43] Zakynthinos GE, Gialamas I, Tsolaki V, Pantelidis P, Goliopoulou A, Gounaridi MI, et al Tailored therapies for cardiogenic shock in hypertrophic cardiomyopathy: navigating emerging strategies. J Cardiovasc Dev Dis 2024;11:401. 10.3390/jcdd11120401 39728291 PMC11678468

[44] Boyd SM, Riley KL, Giesinger RE, McNamara PJ. Use of vasopressin in neonatal hypertrophic obstructive cardiomyopathy: case series. J Perinatol 2021;41:126–33. 10.1038/s41372-020-00824-7 32951013

[45] Balik M, Novotny A, Suk D, Matousek V, Maly M, Brozek T, et al Vasopressin in patients with septic shock and dynamic left ventricular outflow tract obstruction. Cardiovasc Drugs Ther 2020;34:685–8. 10.1007/s10557-020-06993-4 32488425

[46] van Diepen S, Katz JN, Albert NM, Henry TD, Jacobs AK, Kapur NK, et al Contemporary management of cardiogenic shock: a scientific statement from the American Heart Association. Circulation 2017;136:e232–68. 10.1161/CIR.0000000000000525 28923988

[47] Di Vece D, Silverio A, Bellino M, Galasso G, Vecchione C, La Canna G, et al Dynamic left intraventricular obstruction phenotype in Takotsubo syndrome. J Clin Med 2021;10:3235. 10.3390/jcm10153235 34362020 PMC8347696

[48] Giuliana C, Filomia S, Ricci M, Pinnacchio G, D'Aiello A, Saponara G, et al Treating the broken heart: role of stellate ganglion block in Takotsubo syndrome. J Cardiovasc Pharmacol 2025;86:234–8. 10.1097/FJC.0000000000001734 40658069

[49] Beneduce A, Bertoldi LF, Melillo F, Baldetti L, Spoladore R, Slavich M, et al Mechanical circulatory support with Impella percutaneous ventricular assist device as a bridge to recovery in Takotsubo syndrome complicated by cardiogenic shock and left ventricular outflow tract obstruction. JACC Cardiovasc Interv 2019;12:e31–2. 10.1016/j.jcin.2018.10.046 30711550

[50] Uribarri A, Vazirani R, Delia MA, Tomasino M, Fernández-Cordón C, Martín A, et al Impact of mechanical circulatory support on outcomes in Takotsubo syndrome complicated by cardiogenic shock: insights from the RETAKO registry. Int J Cardiol 2025;419:132681. 10.1016/j.ijcard.2024.132681 39454688

[51] Castagna F, Mehra MR, Nabzdyk CGS, Givertz MM. Vasoplegia syndrome after cardiac surgery: insights into mechanisms and treatment. JACC Heart Fail 2025;13:102482. 10.1016/j.jchf.2025.02.028 40466258

[52] Duchnowski P, Śmigielski W. Usefulness of myocardial damage biomarkers in predicting cardiogenic shock in patients undergoing heart valve surgery. Kardiol Pol 2024;82:423–6. 10.33963/v.phj.99553 38493460

[53] Soar J, Berg KM, Andersen LW, Böttiger BW, Cacciola S, Callaway CW, et al Adult advanced life support: 2020 international consensus on cardiopulmonary resuscitation and emergency cardiovascular care science with treatment recommendations. Resuscitation 2020;156:A80–A119. 10.1016/j.resuscitation.2020.09.012 33099419 PMC7576326

[54] Soar J, Böttiger BW, Carli P, Couper K, Deakin CD, Djärv T, et al European Resuscitation Council Guidelines 2021: adult advanced life support. Resuscitation 2021;161:115–51. 10.1016/j.resuscitation.2021.02.010 33773825

[55] Holmberg MJ, Issa MS, Moskowitz A, Morley P, Welsford M, Neumar RW, et al Vasopressors during adult cardiac arrest: a systematic review and meta-analysis. Resuscitation 2019;139:106–21. 10.1016/j.resuscitation.2019.04.008 30980877

[56] Finn J, Jacobs I, Williams TA, Gates S, Perkins GD. Adrenaline and vasopressin for cardiac arrest. Cochrane Database Syst Rev 2019;1:CD003179. 10.1002/14651858.CD003179.pub2 30653257 PMC6492484

[57] Stub D, Bernard S, Duffy SJ, Kaye DM. Post cardiac arrest syndrome: a review of therapeutic strategies. Circulation 2011;123:1428–35. 10.1161/CIRCULATIONAHA.110.988725 21464058

[58] Jentzer JC, Ternus B, Eleid M, Rihal C. Structural heart disease emergencies. J Intensive Care Med 2021;36:975–88. 10.1177/0885066620918776 32314662

[59] Persichini R, Silva S, Teboul JL, Jozwiak M, Chemla D, Richard C, et al Effects of norepinephrine on mean systemic pressure and venous return in human septic shock. Crit Care Med 2012;40:3146–53. 10.1097/CCM.0b013e318260c6c3 22926333

[60] Dayer N, Ltaief Z, Liaudet L, Lechartier B, Aubert JD, Yerly P. Pressure overload and right ventricular failure: from pathophysiology to treatment. J Clin Med 2023;12:4722. 10.3390/jcm12144722 37510837 PMC10380537

[61] Baldetti L, Pagnesi M, Gramegna M, Belletti A, Beneduce A, Pazzanese V, et al Intra-aortic balloon pumping in acute decompensated heart failure with hypoperfusion: from pathophysiology to clinical practice. Circ Heart Fail 2021;14:e008527. 10.1161/CIRCHEARTFAILURE.121.008527 34706550

[62] Rohm CL, Gadidov B, Ray HE, Mannino SF, Prasad R. Vasopressors and inotropes as predictors of mortality in acute severe cardiogenic shock treated with the Impella device. Cardiovasc Revasc Med 2021;31:71–5. 10.1016/j.carrev.2020.12.001 33309042

[63] Millar JE, Fanning JP, McDonald CI, McAuley DF, Fraser JF. The inflammatory response to extracorporeal membrane oxygenation (ECMO): a review of the pathophysiology. Crit Care 2016;20:387. 10.1186/s13054-016-1570-4 27890016 PMC5125043

[64] Klein T, Grandmougin D, Liu Y, Orlowski S, Albuisson E, Tran N, et al Comparison of vasopressin versus norepinephrine in a pig model of refractory cardiogenic shock complicated by cardiac arrest and resuscitated with veno-arterial ECMO. Shock 2021;56:473–8. 10.1097/SHK.0000000000001747 33555846

